# Development and Characterization of Hybrid Meat Analogs from Whey Protein-Mushroom Composite Hydrogels

**DOI:** 10.3390/gels10070446

**Published:** 2024-07-05

**Authors:** Ramdattu Santhapur, Disha Jayakumar, David Julian McClements

**Affiliations:** 1 Biopolymers and Colloids Laboratory, Department of Food Science, University of Massachusetts, Amherst, MA 01003, USA; rsanthapur@umass.edu (R.S.); djayakumar@umass.edu (D.J.); 2 Department of Food Science & Bioengineering, Zhejiang Gongshang University, 18 Xuezheng Street, Hangzhou 310018, China

**Keywords:** whey protein isolate, mushrooms, rheology, hybrid foods, sustainable foods

## Abstract

There is a need to reduce the proportion of animal-derived food products in the human diet for sustainability and environmental reasons. However, it is also important that a transition away from animal-derived foods does not lead to any adverse nutritional effects. In this study, the potential of blending whey protein isolate (WPI) with either shiitake mushroom (SM) or oyster mushroom (OM) to create hybrid foods with enhanced nutritional and physicochemical properties was investigated. The impact of OM or SM addition on the formation, microstructure, and physicochemical attributes of heat-set whey protein gels was therefore examined. The mushroom powders were used because they have relatively high levels of vitamins, minerals, phytochemicals, and dietary fibers, which may provide nutritional benefits, whereas the WPI was used to provide protein and good thermal gelation properties. A variety of analytical methods were used to characterize the structural and physicochemical properties of the WPI-mushroom hybrids, including confocal microscopy, particle electrophoresis, light scattering, proximate analysis, differential scanning calorimetry, thermogravimetric analysis, dynamic shear rheology, textural profile analysis, and colorimetry. The charge on whey proteins and mushroom particles went from positive to negative when the pH was raised from 3 to 9, but whey protein had a higher isoelectric point and charge magnitude. OM slightly increased the thermal stability of WPI, but SM had little effect. Both mushroom types decreased the lightness and increased the brownness of the whey protein gels. The addition of the mushroom powders also decreased the hardness and Young’s modulus of the whey protein gels, which may be because the mushroom particles acted as soft fillers. This study provides valuable insights into the formation of hybrid whey protein-mushroom products that have desirable physiochemical and nutritional attributes.

## 1. Introduction

Recently, there has been considerable concern about the adverse effects of livestock production on the environment, including relatively high levels of greenhouse gas emissions, pollution, freshwater use, land use, and biodiversity [1,2]. For this reason, there has been interest in switching to a diet that includes alternative sources of protein, such as those derived from plants, insects, fungi, precision fermentation, or cultured animal cells [3,4]. However, it is often challenging to simulate the desirable sensorial and nutritional attributes of animal-derived products, like meat, egg, or dairy products, using a single source of alternative proteins [5,6]. In particular, it has proved challenging to accurately mimic the appearance, texture, mouthfeel, flavor, and nutrient profile of animal-derived products using only a single type of alternative proteins. For this reason, there is interest in creating hybrid food products that combine protein-rich ingredients from different sources, such as animals, plants, insects, fungi, precision fermentation, or cultured animal cells, to enhance the sensory and nutritional properties of foods, while also improving their sustainability [7,8,9]. These hybrid products can reduce the proportion of animal-derived foods in the human diet, while still having beneficial sensorial and nutritional attributes. Nevertheless, the types and amounts of the different types of ingredients used to formulate a hybrid food must be optimized to obtain a final product with the desired properties.

In this study, we focused on creating meat analogs by fabricating hybrid foods consisting of milk proteins and powdered mushrooms. Whey protein isolate (WPI) is a mixture of globular proteins that are normally isolated and purified from the whey fraction of milk [10]. Powdered WPI is used as a multifunctional ingredient in the food industry due to its good gelling, binding, foaming, emulsifying, and nutritional properties [11]. Unlike many plant proteins, whey protein has adequate levels of all the essential amino acids required to maintain human health and wellbeing [12]. One of the most important functional attributes of whey proteins are their ability to form irreversible heat-set gels when heated above their thermal denaturation temperature [10]. Under these conditions, the globular whey proteins unfold and expose non-polar groups on their surfaces, which strengthens the hydrophobic attraction between them. As a result, they may associate with each other and form a 3D network mainly held together by hydrophobic, disulfide, and hydrogen bonding [13]. The microstructure, appearance, rheology, and fluid holding properties of these gels are strongly influenced by protein concentration, as well as pH, ionic strength, and heating conditions, as these factors influence the nature of the aggregates formed [14,15]. Whey proteins are therefore useful functional ingredients for creating nutritious foods with textural attributes that can somewhat mimic those of meat products [16,17]. Nevertheless, the raising of dairy cattle to produce milk is still damaging to the environment [18], and so it would be advantageous to reduce the amounts of whey protein used in these products. Moreover, many bioactive substances are lacking from whey proteins, such as dietary fibers and nutraceuticals.

Mushrooms are a sustainable and nutritious food source that are rich in health-promoting bioactive substances [19,20]. Shiitake mushrooms (*Lentinula edodes*) are one of the most common types of edible mushrooms consumed commercially [21]. They have a high nutritional value because they are abundant in vitamins (niacin, pro-vitamin D_2_, vitamin B_1_, B_2_, B_6_, and B_12_) and minerals (potassium, manganese, magnesium, iron, and phosphorus), as well as bioactive substances such as dietary fiber, ergosterol, and natural antioxidants [22]. Oyster mushrooms (*Pleurotus ostreatus*) are also commonly consumed around the world because of their desirable flavor, nutrient content, and therapeutic qualities. Indeed, they have been reported to exhibit antidiabetic [23], antibacterial [24], antiviral [25], antioxidant [26], anticancer [27], and cholesterol-lowering properties [28], which have been attributed to the broad range of bioactive substances they contain [26]. If not consumed directly, mushrooms can be converted into a powdered form, which reduces waste, extends their shelf life, and facilitates storage and transport [29]. These powdered mushroom ingredients may be useful additives for improving nutritional properties and altering the physicochemical and functional properties of whey proteins. Researchers have shown that incorporating powdered mushrooms into meat emulsions enhanced their nutritional profile, promoted the formation of a finer emulsion, and increased gel strength [30]. Other researchers reported that the incorporation of powdered mushrooms into a fish meat formulation could inhibit lipid oxidation due to the antioxidant agents within the mushrooms [30]. Other researchers have examined the impact of mushroom powder addition on the properties of chicken [31], beef [32], pork [33], and goat [34] products. Due to their desirable nutritional, sensory, and functional attributes, mushroom powders are particularly suitable for formulating hybrid meat analogs [35].

The objective of this research was therefore to characterize the impact of shiitake mushroom (SM) and oyster mushroom (OM) powders on the structural and physicochemical properties of whey protein gels. It was hypothesized that incorporation of the mushroom powders would alter the appearance, texture, and fluid holding properties of the whey protein gels, which may be beneficial for creating hybrid meat analogs. Moreover, the incorporation of the mushroom powders should improve the nutritional and sensory attributes of the meat analogs [36]. For this reason, we examined the impact of incorporating SM or OM into whey protein gels on their structural and physicochemical properties using a variety of analytical methods, including microscopy, calorimetry, gravimetry, electrophoresis, rheology, and colorimetry. The knowledge gained from this study may facilitate the development of hybrid alternative protein products with enhanced sensorial and nutritional attributes.

## 2. Results and Discussion

### 2.1. Mushroom Powder Composition

The proximate compositions of the two mushroom samples used in the current study were the same as those reported in our previous research [37]. Briefly, the carbohydrate, protein, moisture, ash, and fat contents of the SM were 67.1, 14.6, 9.8, 5.6, and 3.0% (*w*/*w*) respectively, while those of the OM were 60.4, 17.1, 11.6, 5.9, and 5.0% (*w*/*w*), respectively. The proximate analysis therefore indicates that the majority of the mushroom powders was carbohydrates, which is a mixture of digestible carbohydrates such as starches, glycogen, and sugars [38], as well as indigestible carbohydrates such as chitin, *β*-glucans, hemicellulose, and pectin [39].

### 2.2. Thermogravimetric Analysis

**Figure 1:**
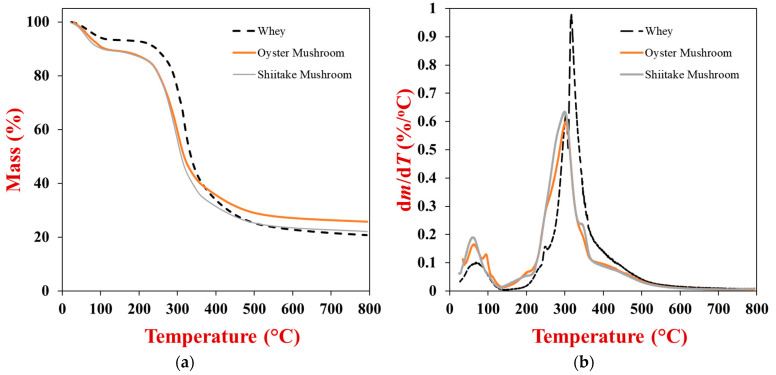
(**a**) Thermogravimetric analysis (TGA) and (**b**) derivative thermogravimetric (DTG) analyses of powdered whey protein, oyster mushroom, and shitake mushroom from 25 to 800 °C during heat at 10 °C/min.

Information about the thermal behavior of the powdered whey protein and mushroom samples was obtained by measuring the decrease in their mass during controlled heating using thermogravimetric analysis (Figure 1a). The gradient of the mass change with temperature change (d*m*/d*T*) was calculated from this data to identify the temperature ranges where the major thermal events occurred (Figure 1b). For all samples, there was a pronounced decrease in mass during heating from around 30 and 120 °C, which can be attributed to evaporation of some of the water molecules associated with the powders. At 120 °C (after this thermal event was complete), the mass loss of the samples was around 6.8, 10.3, and 10.5% (*w*/*w*) for the WPI, OM, and SM powders, respectively, which was probably because they initially contained different amounts of water. There was another pronounced decrease in mass for all samples between 210 and 360 °C, which was probably due to thermal degradation of organic compounds, such as proteins, carbohydrates, and other substances. Indeed, other researchers have reported that WPI undergoes thermal decomposition over a similar temperature range, which was attributed to breakage of covalent peptide bonds and cleavage of S-S, O-N, and O-O bonds at higher temperatures [40]. Other researchers have reported similar TGA curves for powdered mushrooms (shitake) analyzed by thermogravimetric analysis, which were also attributed to moisture evaporation at lower temperatures and thermal degradation of organic matter at higher temperatures [41]. At the end of the TGA experiments (800 °C), there were slight differences in the residual masses: 19.5, 25.8, and 22.1% (*w*/*w*) for the WPI, OM, and SM powders, respectively. This effect may have been because they contained different kinds of minerals and mineral compounds formed during the heating process, as these components have a low volatility and therefore tend to remain after the samples have been heated at high temperatures [42].

### 2.3. Zeta-Potential Analysis

**Figure 2:**
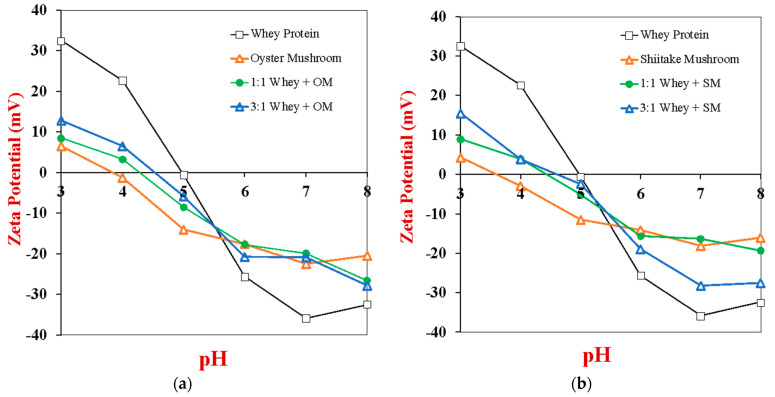
Impact of pH on the zeta potential of 0.1% (*w*/*v*) whey protein, 0.1% (*w*/*v*) mushroom and 0.1% (*w*/*v*) whey protein-mushroom (1:1 and 3:1) dispersions: (**a**) oyster mushroom; (**b**) shitake mushroom.

The whey protein and/or mushroom powders were then dispersed in aqueous solutions and their electrical characteristics were determined using electrophoresis (Figure 2). For the WPI dispersion, the zeta potential changed from around +20 mV at pH 3 to −35 mV at pH 8, with a point of zero charge around pH 5. The pH dependent charge of the WPI dispersion is because the ionizable surface groups on the whey protein surfaces change from protonated (−NH_3_^+^ and −COOH) at low pH values to de-protonated (−NH_2_ and −COO^−^) at high pH values [43]. At the isoelectric point, there is a balance of charged amino and carboxyl groups, leading to a zero net charge [44]. For the mushroom dispersions (OM and SM), their zeta potential values also went from positive at low pH to negative at high pH, however, there were some distinct differences in their pH behavior compared to the whey proteins. First, the magnitude of their zeta potential was much lower than that of the whey proteins at both low pH values and high pH values. For instance, the zeta potential of the OM and SM samples was only +6.5 and +4.4 mV at pH 3, and −20.5 and −16.1 mV at pH 7, respectively. Second, the point of zero charge for the mushroom samples was appreciably below that of the whey proteins. For instance, the isoelectric points of the OM and SM samples were around pH 3.9 and 3.6, respectively. There are several possible reasons for the different zeta potential-pH behaviors of the whey proteins and mushroom particles. The WPI powder is mainly composed of pure proteins, whereas the mushroom powders contain a complex mixture of carbohydrates, proteins, lipids, and minerals. Any anionic polysaccharides (like pectin) or cationic polysaccharides (like chitosan) present in the mushrooms would have interacted with oppositely charged groups on the amphoteric proteins, thereby partially neutralizing their charge [45,46].

The zeta potential *versus* pH profiles of the hybrid samples containing 1:1 or 1:3 WPI-to-mushroom, were between those of the pure WPI and pure mushroom for both OM and SM. As expected, the zeta potential and point of zero charge values became closer to that of pure mushroom as the OM or SM content increased. Interestingly, however, the zeta potential values of the 1:1 hybrids were much closer to those of the pure mushroom than to those of the pure WPI. There are several potential reasons for this: The mushroom powders contained a significant amount of minerals, which may have reduced the magnitude of the zeta potential on the proteins through electrostatic screening and/or ion binding effects. Moreover, the mushroom powders contained other components (such as ionic dietary fibers) that may have interacted with the proteins. In addition, the whey proteins and mushroom particles may have formed complexes under some pH conditions [45,46]. 

Under the solution conditions used to form the heat-set gels in this study (pH 7.0), both the whey proteins and mushroom powders would be expected to be negatively charged. Consequently, there should be an electrostatic repulsion between them, which may impact their ability to form hybrid gels. 

### 2.4. Differential Scanning Calorimetry Analysis

**Figure 3:**
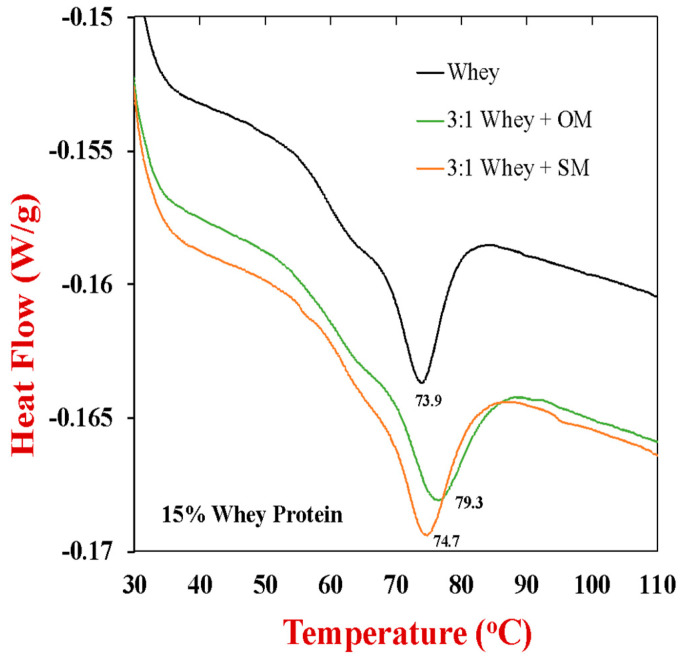
Differential scanning calorimetry profile of 15 wt% whey protein solutions in the absence and presence of 5 wt% mushroom (oyster or shitake) during heating from 25 to 150 °C with at 3 °C/min.

The impact of the mushroom powders on the thermal stability of the whey proteins was then analyzed using DSC, as the unfolding and aggregation of globular proteins during heating is critical for the creation of heat-set gels. These measurements were carried out for WPI solutions (10, 15, 20%), mushroom solutions (5% and 10%), and hybrid solutions (1:1 or 3:1, 20% total). For the sake of clarity, we only show the results for the samples containing 15% WPI with or without 5% mushroom (SM or OM) in Figure 3. For the pure WPI solutions (15%), a single endothermic peak was observed around 73.9 °C, which can be attributed to irreversible denaturation and aggregation of the globular proteins (mainly *β*-lactoglobulin) around this temperature [47,48]. The endothermic enthalpy change is a result of the energy required to disrupt the intramolecular bonds that hold the globular proteins together in their native state, such as hydrophobic, hydrogen, and disulfide bonds [49]. No thermal transitions were observed in the SM or OM samples during heating, which may have been because their protein content was too low, or because the protein was already denatured. It is possible that the procedures used to dehydrate the mushrooms and convert them into a powdered form promoted some unfolding of their proteins. The presence of the shitake mushroom powder caused little change in the thermal denaturation temperature of the whey proteins (74.7 °C). Conversely, the presence of the oyster mushroom powder caused an appreciable rise in the thermal denaturation temperature of the whey proteins (79.3 °C). Similar results were obtained for the samples containing 10% WPI with and without 10% mushroom, with the thermal denaturation temperature changing from around 75.1 °C in the absence of mushroom to 76.3 and 80.9 °C in the presence of 10% SM and OM, respectively. These results suggest that some of the constituents within the oyster mushrooms may have interacted with the whey proteins and enhanced their thermal stability. 

### 2.5. Dynamic Shear Rheology Analysis

**Figure 4:**
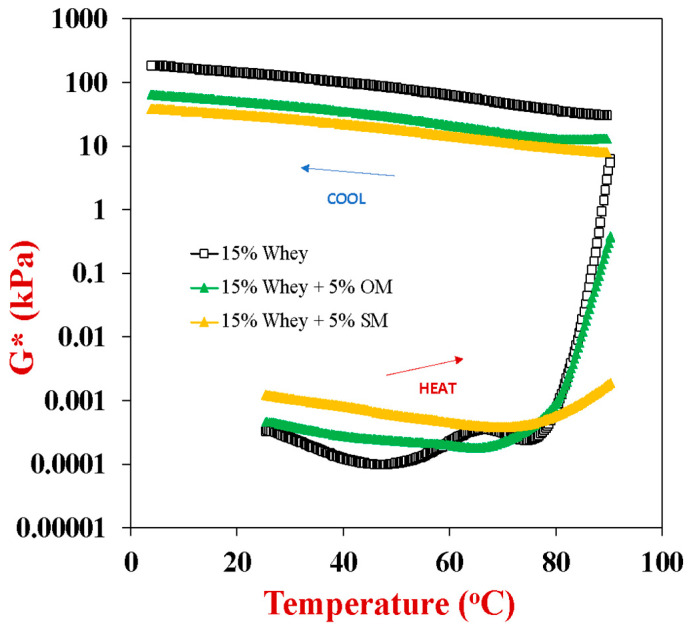
Temperature dependence of the complex shear modulus (G*) of 15% whey protein solutions in the absence and presence of 5% oyster or shitake mushroom (OM or SM). The samples were heated from 25 to 90 and then cooled from 90 to 5 °C at 3 °C/min. A constant frequency (1 Hz) and strain (0.1%) were used. All solutions contained 100 mM NaCl.

The dynamic shear rheology of the whey protein and/or mushroom samples were measured as a function of temperature (Figure 4). For the sake of clarity, we only show the results for the samples containing 15% whey protein with or without 5% mushroom powder (SM or OM). For the pure WPI samples, the complex shear modulus *versus* temperature profiles showed that the protein formed viscous solutions before heating. During heating from 25 to 50 °C, there was a decrease in shear modulus, which may have been due to dissociation of whey protein aggregates or oligomers. From 50 to 65 °C, there was a slight increase in shear modulus, which can be attributed to unfolding and aggregation of the more thermally labile whey proteins. WPI ingredients contain a mixture of globular proteins with different denaturation temperatures, including *β*-lactoglobulin (72 °C), α-lactalbumin (64 °C), and bovine serum albumin (64 °C) [50]. As they are typically present at the highest concentration in whey proteins, they led to a stronger gel network being formed. The shear modulus of the pure WPI gels increased when they cooled, which can mainly be attributed to strengthening of the hydrogen bonding between the protein molecules in the gel network. These results show that the pure whey protein formed irreversible heat-set gels. 

The pure mushroom dispersions (5 or 10%) did not form strong gels upon heating. The 5% OM and SM dispersions could not be analyzed because the mushroom particles sedimented to the bottom of the samples during the rheology measurements. In contrast, the 10% OM and SM dispersions could be analyzed because they formed weak gels that were resistant to sedimentation. However, their gel strength did not change appreciably during heating or cooling, as reported in our previous study [37]. This result suggests there were no components within the pure mushroom dispersions (such as proteins or polysaccharides) that underwent thermal transitions and formed strong gel networks. These results are therefore consistent with the DSC measurements discussed earlier, which also detected no thermal transitions within the mushroom samples during heating and cooling (Section 2.4).

The presence of the mushroom powders altered the thermal gelation behavior of the whey proteins. Before heating, the initial shear modulus of the hybrid samples was higher than that of the pure protein samples, which was attributed to the formation of a weak gel network by the mushroom particles at ambient temperature. In the presence of proteins, this network was relatively resistant to sedimentation and so rheology measurements could be performed. For oyster mushrooms, the shear modulus decreased slightly from 25 to 65 °C, but then increased steeply from 70 to 90 °C, which can be attributed to unfolding and aggregation of the whey protein molecules. In contrast, for the shitake mushrooms, the shear modulus decreased slightly from 25 to 75 °C, increased moderately from 75 to 90 °C, and then continued to increase rapidly when the sample was held at 90 °C. This result suggests that the presence of the SM inhibited the unfolding and/or aggregation of the whey proteins. The DSC results suggested that the presence of OM increased the thermal denaturation temperature of the whey proteins, whereas the presence of SM had little effect (Figure 3). Taken together, these results therefore suggest that SM inhibited protein aggregation rather than protein unfolding. During cooling, the shear modulus of both types of hybrid gels increased, which can again be attributed to strengthening of hydrogen bonding between the biopolymers at lower temperatures. The final shear modulus of the hybrid gels was lower than that of the whey protein gels. For instance, the final shear modulus values were 186, 65, and 38 kPa for the 15% WPI, 15%WPI + 5%OM, and 15%WPI + 5%SM samples at 5 °C, respectively. This result also suggests that the presence of the SM inhibited the aggregation of the whey protein molecules, thereby leading to a weaker gel network being formed. The samples containing 10% whey protein and 10% mushroom also showed weaker gel strengths than the ones containing only 10% whey protein. In this case, the final shear modulus values were 39, 5.8, and 17 kPa for the 10% WPI, 10%WPI + 10%OM, and 10%WPI + 10%SM samples at 5 °C, respectively. Thus, the presence of the OM led to weaker gels than the presence of SM for these samples. The origin of this difference is unknown, but our results clearly show that the mushroom particles interfered with the ability of the whey proteins to form gels.

### 2.6. Texture Profile Analysis

**Figure 5:**
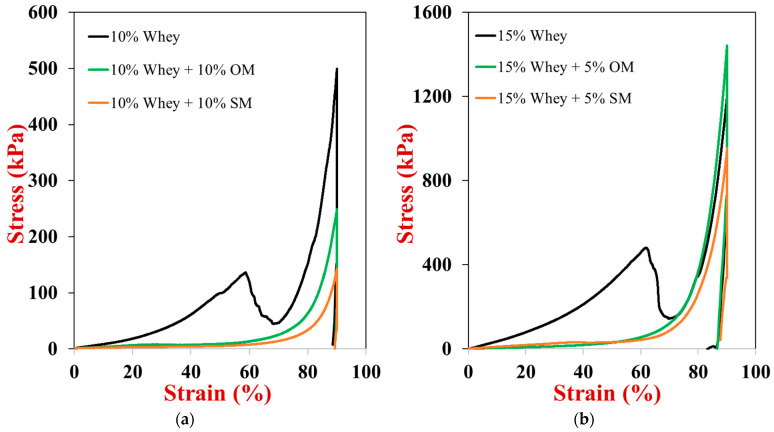
Effect of single composition on the stress-strain relationship of whey protein and whey protein-mushroom hybrids during single compression-decompression experiments (25 °C) (**a**) 10% whey protein with or without 10% mushroom (**b**) 15% whey protein with or without 5% mushroom.

Further insights into the impact of the mushroom powders on the thermal gelation properties of the whey proteins were obtained using compression testing. Examples of the single compression stress versus strain curves for the 10% and 15% (*w*/*w*) whey protein gels with and without mushroom (to bring the total to 20% *w*/*w*) are shown in Figure 5. The Young’s modulus, breaking stress, and breaking strain calculated from these curves for all the samples tested are summarized in Table 1. For both the pure WPI gels, the stress increased with increasing strain until the samples were compressed to around 60% of their original height, and then there was a steep decrease in stress, which was taken to be the breaking point. For these gels, the Young’s modulus, breaking stress, and breaking strain increased as the protein concentration was increased. The strengthening of the WPI gels with increasing protein concentration is because more denatured protein molecules can be incorporated into the gel network during heating, leading to more crosslinks per unit surface area, and therefore a greater resistance to compression and fracture.

Interestingly, the samples containing mushroom powder were much softer than the pure WPI gels with the same protein content, and they did not exhibit a breaking point over the range of strains used, i.e., 0 to 90% (Figure 5). Consistent with the complex shear modulus measurements, the Young’s modulus of the hybrid gels was considerably lower than that of the pure WPI gels at the same protein content, which suggests that the presence of the mushroom particles interfered with the ability of the unfolded whey proteins to associate with each other and form a gel network. However, the impact of the mushrooms on gel strength depended on mushroom type. For example, at 10% WPI, the Young’s modulus of the gels decreased in the following order WPI >> WPI + OM > WPI + SM but at 15% WPI, the Young’s modulus decreased in the following order WPI >> WPI + SM > WPI + OM. These trends are therefore the same as those observed for the dynamic shear modulus experiments discussed in Section 2.5. Interestingly, the incorporation of the mushroom powders into the WPI gels increased both their breaking stress and breaking strain. Consequently, the hybrid gels were much more resistant to rupture during compression than that of pure WPI gels. This may be an important attribute for some kinds of plant-based foods.

A double compression test was then used to provide additional insights into the impact of the mushroom powders on the textural attributes of the WPI gels. The force *versus* distance profiles of the WPI and the hybrid gels were measured as they were subjected to two successive compression-decompression cycles. The TPA parameters were then calculated from these profiles and reported in Table 2. For the pure WPI gels, the hardness, springiness, gumminess, and chewiness increased with increasing protein concentration, which was probably because more protein molecules were incorporated into the gel network. The addition of the mushroom powders had a major impact on the TPA parameters of the whey protein gels, with the effects depending on gel composition. For both mushroom types, the hardness, adhesiveness, and gumminess of the hybrid gels were considerably higher than those of the pure WPI gels. The increase in hardness may be because the hybrid samples did not fracture during the first compression, and therefore provided a greater resistance to compression at the higher strains used. For both protein concentrations, the addition of the OM gave higher hardness values than the addition of SM. The increase in the adhesiveness and gumminess of the gels after the addition of the mushroom powders suggests that there were some components in the mushrooms that increased their ability to stick to surfaces, such as starches or dietary fibers. 

### 2.7. Tristimulus Color Analysis

**Figure 6:**
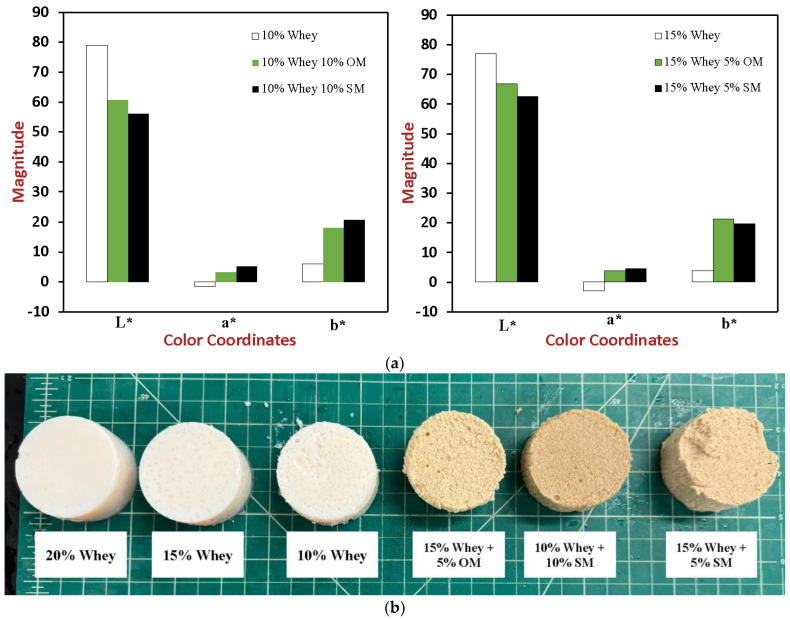
(**a**). Tristimulus color coordinates (L* a* b*) and (**b**) digital photographs of whey protein and whey protein-mushroom hybrid gels with different compositions (25 °C).

The tristimulus color coordinates (L*, a*, and b*) of the samples were measured to assess the impact of the two different mushroom powders on the appearance of the heat-set WPI gels (Figure 6). The lightness of the pure WPI gels was relatively high and decreased with increasing protein content, being 79.0, 76.9, and 73.0 for the samples containing 10%, 15%, and 20% *w*/*w* protein, respectively. Moreover, these samples became slightly more green and slightly less yellow with increasing protein, with the a* values being −1.6, −3.1, and −3.7, and the b* values being +5.8, +3.8, and +2.7 for WPI gels containing 10%, 15%, and 20% *w*/*w* protein, respectively. These effects may be a result of changes in the selective absorption and scattering of light waves by the samples with increasing protein content [50]. The whey protein powder had a slightly beige color, indicating that it contained some pigments that could selectively absorb light. Under the solution conditions used, the WPI gels would have contained particulate protein aggregates [15], which would scatter light waves by an amount depending on their size, shape, and packing. The protein content would be expected to impact the nature of the particulate aggregates formed, and therefore the color coordinates of the heat-set WPI gels. The surface smoothness of the WPI gels increased with increasing protein content, as seen in the digital photographs of them (Figure 6b). 

The addition of mushroom powders to the WPI gels had a major impact on their tristimulus color coordinates (Figure 6a). In general, the lightness of the hybrid gels was lower than that of the WPI gels, and their color was stronger. Specifically, the samples changed from greenish (−a*) to reddish (+a*) after addition of the mushroom powders, they became more yellowish (higher +b values). The decrease in lightness and change in color of the WPI gels after addition of the mushroom powders can mainly be attributed to the fact that they contained natural pigments that could selectively absorb light. Indeed, the mushroom powders had a brownish color. The impact of the mushroom powders on the overall appearance of the gels was assessed by taking digital photographs of them (Figure 6b). These photographs clearly showed that the hybrid gels had a darker appearance than the WPI gels. They also showed that the hybrid gels had a much rougher surface texture, which suggests that the presence of the mushroom particles disrupted the 3D gel network normally formed by the whey proteins.

The brownish color, softer texture, and rougher surface texture of the hybrid gels may be useful for some food applications. For instance, they may be useful for creating pate type products that replace meat-based ones. 

### 2.8. Water Holding Capacity Analysis

The ability of foods (like meat analogs) to retain fluids plays an important role in their texture and mouthfeel [51]. For this reason, the water holding capacity of heat-set gels prepared using different amounts of whey protein and/or mushroom powders were measured. The WHC values of all the samples was very high (>99.5%), suggesting that they retained fluids well. This effect was probably because the whey proteins formed a 3D biopolymer network that was relatively strong and contained small pores [52]. As a result, there were strong capillary forces holding the water within the biopolymer network, and the network did not collapse during centrifugation, thereby leading to a high WHC [53,54]. The good fluid holding properties of whey-mushroom hybrid products may be useful for some commercial applications.

### 2.9. Microstructure Analysis

**Figure 7:**
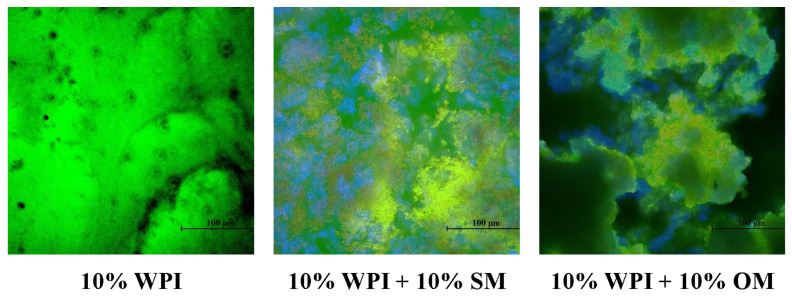
Confocal microscopy images of whey protein and whey protein-mushroom hybrids with different compositions (25 °C).

Confocal fluorescence microscopy with selective staining was used to characterize the microstructure of the heat-set whey protein and hybrid gels. Lipid-rich areas were stained red, polysaccharides blue, and proteins green, and microscopy images, as shown in Figure 7, were only obtained for samples containing 10% WPI, with or without 10% mushroom, due to the difficulty in preparing other samples for analysis. Significant differences between the hybrids and pure WPI samples were observed. The 10% WPI samples appeared green throughout, which indicates that a protein gel network extended throughout these samples, however, there was evidence of some large protein clumps. The samples containing 10% WPI and 10% mushroom had a much more heterogeneous appearance, with a green (protein) network and a blue (polysaccharide) network being observed. There were also many large dark regions present in the hybrid gels (especially the ones containing OM), which may have been voids or fibrous areas that were too dense for the dye to penetrate. These results suggest that the presence of the mushroom powders disrupted the normal 3D network formed by the whey protein molecules, which may account for their ability to reduce the shear and Young’s modulus of the hybrid gels.

## 3. Conclusions

In conclusion, this work has shown that adding shitake or oyster powder to whey protein gels changed their rheological and optical characteristics. Compared to pure whey protein gels, they produced gels that were significantly softer, more malleable, and browner. The physicochemical origin of these effects is currently not fully understood. The pigments and particles in the powdered mushrooms change how light waves are selectively absorbed and scattered by the gels, thereby changing the color and lightness of the gels. A variety of components in the powdered mushrooms may modulate the way the protein molecules interact with each other and form three-dimensional networks. For instance, mineral ions from the mushrooms may be involved in ion binding and electrostatic screening effects, which may alter the electrostatic interactions between the proteins. Additionally, the mushrooms contain polysaccharides (dietary fibers and starches) that can thicken the aqueous phase surrounding the proteins, thereby slowing down their movement and interactions, which could inhibit the formation of a strong gel network. Additionally, some of the polysaccharides may bind to the whey proteins or cause phase separation, which could also alter protein gelation. Lastly, the hydrated particles in the powdered mushrooms would act as “soft” fillers if their elastic modulus was less than that of the whey protein gel matrix surrounding them. As a result, they would have reduced the stiffness of the hybrid gels. Further research is clearly needed to elucidate the molecular and physicochemical origin of the influence of mushroom particles on the physicochemical characteristics of hybrid whey protein-mushroom gels.

The findings of this study show that hybrid WPI-mushroom gels with a variety of physicochemical characteristics can be created by varying their composition. Because the hybrid gels contain dietary fibers, vitamins, minerals, and bioactive components from the mushrooms, their nutritional profiles should be better than those of pure whey protein gels. Furthermore, the potential to produce hybrid gels with various textures and appearances may increase the number of applications for whey proteins as useful components in more environmentally friendly and sustainable food products. For example, some of the hybrid gels that were generated were brownish in color, moderately soft, and bendable, which could make them good for creating spreadable meat paste analogs. However, animal meat is known for its characteristic tenderness, juiciness, and flavor, influenced in part by the type and quantity of fat present. In future studies, it would therefore be useful to examine the impact of incorporating lipids into the WPI-mushroom gels on their appearance, texture, stability, and nutritional profile.

## 4. Materials and Methods

### 4.1. Materials

Dried oyster and shiitake mushrooms were purchased from New Tiger International Company (Westbury, NY, USA). Sodium chloride (NaCl) was purchased from the Sigma-Aldrich Co., Ltd. (St. Louis, MO, USA). Whey protein isolate (BiPro) was obtained from Davisco Foods International, Inc. (Eden Prairie, MN, USA).

### 4.2. Sample Preparation 

#### 4.2.1. Mushroom Powder Preparation

The dried mushroom powders were initially sheared using a high-speed blender for 90 s at 6000 rpm to reduce their particle size and improve their homogeneity. The finely ground mushroom powders were then sieved using a 20 mm mesh screen to remove any larger particles.

#### 4.2.2. Protein and Protein-Mushroom Dispersion and Gel Preparation

Whey protein isolate and mushroom powders were mixed in different ratios and then dispersed in double distilled water and stirred overnight at 350 rpm using a magnetic stirrer to hydrate and disperse them. Samples were prepared consisting of 10%, 15%, and 20% WPI only, as well as 10% WPI and 10% mushroom (10:10), and 15% WPI and 5% mushroom (15:5). These compositions were selected because they could form heat-set gels. In some experiments, these protein and protein-mushroom dispersions were used directly, such as zeta-potential, differential scanning calorimetry, and dynamic shear rheology analyses. In other experiments, they were converted into heat-set gels before analysis by placing them in glass beakers and covering with silver foil followed by heating them in a water bath at 90 °C for 30 min, such as the texture profile, water holding capacity, and microscopy analyses. 

### 4.3. Mushroom Powder Composition

The proximate analysis of the mushroom samples was conducted according to the methods described in detail by the Association of Official Analytical Chemists [55]. The moisture content of the samples was determined using a hot air oven by drying the samples at 100 ± 5 °C to a constant weight. The crude fat content was estimated using a cold extraction method with hexane followed by drying in a hot air oven. The protein content was estimated using the Dumas method using a nitrogen conversion factor for mushrooms of 4.38 [56]. This value is lower than the usual conversion factor for proteins because mushrooms have a substantial amount of non-protein nitrogenous substances (such as chitin). The total ash content was determined by incineration at 550 ± 5 °C using a muffle furnace. Samples that had been previously dried in a hot air oven were used to conduct the ash content analysis. The total carbohydrate content was expressed as the result obtained after subtracting the sum of the moisture, ash, fat, and protein contents from 100. 

### 4.4. Thermogravimetric Analysis

Thermogravimetric analysis (TGA) was carried out on powdered WPI, OM, and SM samples. About 30–40 mg of the powdered samples were loaded into aluminum sample pans (to about 2/3rd full) and then placed within the TGA instrument (Simultaneous Thermal Analyzer SDT 650, TA Instruments, New Castle, DE, USA). Each sample was initially equilibrated at 25 °C and then heated from 25 to 800 °C at 10 °C/min. The change in the mass of the samples with temperature was then recorded during heating [57]. Temperature ranges where major thermal transitions occurred were established by plotting the derivative thermogravimetry (DTG) profile of the mass v*ersus* temperature (d*m*/d*T*) results.

### 4.5. Zeta-Potential Analysis

The electrical characteristics of whey protein molecules, mushroom particles, and their combinations were measured using microelectrophoresis (Zetasizer Nano ZS, Malvern Instruments, Malvern, Worcestershire, UK). Initially, 0.1% (*w*/*v*) SM, OM, and/or WPI dispersions were prepared with pH values ranging from 3 to 8. At least four measurements were made at each data point to ensure accuracy. The zeta-potential of these dispersions was then measured. HCl and NaOH stock solutions were used to make the pH adjustments of the samples. 

### 4.6. Differential Scanning Calorimetry 

Differential Scanning Calorimetry (DSC) was used to assess any thermal transitions of the globular proteins in the samples during heating. Samples (50–70 mg) of protein, mushroom, or protein-mushroom dispersions were placed into high volume aluminum pans, weighed accurately, and then sealed hermetically to prevent loss during the measurement. The sample cells were then placed inside the DSC instrument (DSC25, TA Instruments, New Castle, DE, USA), equilibrated to 25 °C, and then heated from 25 to 110 °C at 3 °C/min, as described previously [57]. Measurements were recorded as the samples were heated and the heat flow (W/g) was plotted against the temperature (°C).

### 4.7. Dynamic Shear Rheology Analysis

Initially, SM, OM, and/or WPI dispersions (pH 7.0, 100 mM NaCl) were prepared by dispersing the powdered ingredients in double distilled water and then stirring overnight at 350 rpm at room temperature. The rheological properties of these different samples were then analyzed under a dynamic shear rheometer (HR20, TA Instruments, New Castle, DE, USA). Around 1.0 to 1.5 mL of the unheated test sample was placed in a parallel plate (40.00 mm, stainless steel, crosshatched, Peltier plate) sample cell. The edges of the sample were then covered with mineral oil and a solvent trap was placed on top of the sample to reduce any evaporation during heating [57]. All analyses were performed using a gap of 1000 μm between the parallel plates. The elastic (G′) and viscous (G″) shear moduli of the samples were then measured as the samples were heated from 25 to 90 °C at 6 °C/min, incubated for 10 min at 90 °C, and then cooled from 90 to 4 °C at 6 °C/min (strain 0.1%, frequency 1 Hz). 

### 4.8. Texture Profile Analysis

Initially, SM, OM, and/or WPI dispersions (pH 7.0, 100 mM NaCl) were prepared by dispersing the powdered ingredients in double distilled water and then stirring overnight at 350 rpm at room temperature. The resulting dispersions were then poured into a glass flask, which was heated by placing it in a water bath at 90 °C for 30 min, followed by cooling in an ice tray for 2 h. The resulting gelled samples were then removed and cut into 1 × 1 × 1 cm^3^ cubes. The textural attributes of the gelled samples were then analyzed using single and double compression tests performed using an instrumental texture analyzer (TA-XT2, Stable Micro System, Surrey, UK):

- Double compression test: The texture profile analysis (TPA) parameters of the samples were determined using a double compression test, which involved a two-cycle compression/decompression program. For these measurements, the pre-test speed, test speed, and post-test speed were set at 2 mm/s, and the final target strain was set at 50%. A 5 s gap separated the two cycles. A cylindrical probe (P/50, 50 mm stainless cylinder) was used to carry out the tests [58]. The hardness, resilience, cohesion, springiness, gumminess, and chewiness of the samples were calculated from the resulting force-distance profiles.
- Single compression test: The Young’s modulus and fracture properties of the samples were assessed using a single compression test. The same probe type was used as for the double compression test. The pre-test speed, test speed, and post-test speed were set to 2, 1, and 10, mm/s, respectively, and the final target strain was set to 90% [57]. The Young’s modulus, fracture stress, and fracture strain were calculated from the resulting stress-strain profiles.

### 4.9. Tristimulus Color Analysis

The tristimulus color coordinates (L*, a*, and b*) were measured for each sample using a colorimeter (ColorFlex EZ 45/0 LAV, Hunter Colorimeter, Hunter, VA, USA). The instrument was first calibrated using standardized white and black plates by measuring the reflectance using a standard light source (D65) and detection angle (10 °C) [39]. During analysis, the samples were placed in thin, transparent containers that were then placed into the measurement cell of the instrument. The samples were covered with a black cup to avoid light contamination during measurements [57]. Digital photographs of the gels were also taken for reference.

### 4.10. Water Holding Capacity Analysis

The water-holding capacity of the thermally induced gels was evaluated in triplicate with slight modifications [59]. Approximately 1 g of various samples, including WPI, OM, SM, WPI + OM, and WPI + SM, were dissolved in double-distilled water containing 100 mM NaCl. The mixtures were then stirred at 350 rpm for 3 h using a magnetic stirrer to hydrate and disperse them. The resulting dispersions were then transferred to 1.5 mL microcentrifuge tubes using a positive-displacement pipette. Following weighing, the tubes were heated at 90 °C for 30 min in a water bath to form heat-set gels. After heating, the samples were cooled to ambient temperature. The inner walls of the tubes were then dapped with a tissue to remove residual moisture and the samples were then weighed. The samples were centrifuged at 300× *g* rpm for 2 min, transferred onto a white sheet for visual inspection, and then allowed to drain by inverting the tubes for 10 min to assess their water content.

### 4.11. Microstructure Analysis

The microstructure of the heat-set protein and/or mushroom gels were observed using a confocal laser scanning microscope with a 10× eye lens and a 40× objective lens (Nikon d-Eclipse C1 80i, Nikon, Melville, NY, USA). Prior to observation, gel samples were cut into thin layers (0.2 mm) using a sharp knife and then placed on the glass slide. The samples were then stained with dyes that were specific for proteins, lipids, or polysaccharides: FITC (1 mg/mL in ethanol) for proteins; Nile red (1 mg/mL in water) for lipids; and calcofluor white (1 mg/mL in ethanol) for polysaccharides [60]. One or two drops of these dye were mixed with the samples and then the mixtures were incubated for 1–2 min. Excess dye was removed using a Kim wipe without disrupting the sample. The samples were then covered with a glass cover slip and observed under a microscope. The images were then collected, stored, and analyzed using the instrument software (NIS-Elements 4.2, Nikon, Melville, NY, USA).

### 4.12. Statistical Analysis

Each experiment was conducted using freshly prepared independent samples in triplicates, except the dynamic shear rheology measurements, which was conducted in duplicates. The results were expressed as means and standard deviations, which were calculated using Microsoft Excel (version 2405 (Build 17628.20144). Significant differences between samples (*p* < 0.05) were determined by performing ANOVA (post hoc Tukey HSD test) using R-program software (R version 4.3.1).

## Figures and Tables

**Table 1:** Young’s modulus, breaking stress and breaking strain of whey protein and whey protein-mushroom samples with different compositions determined using a single compression test. All samples contained 100 mM NaCl.

Group	Young’s Modulus (kPa)	Breaking Stress (kPa)	Breaking Strain (%)
20% Whey	958.85 ± 14.47 ^a^	1035 ± 120 ^b^	66.87 ± 2.9 ^b^
15% Whey	407.78 ± 22.17 ^b^	447 ± 70 ^c^	60.54 ± 2.2 ^c^
10% Whey	105.63 ± 17.38 ^c^	135 ± 30 ^d^	56.26 ± 4.5 ^c^
15% Whey + 5% OM	38.57 ± 16.14 ^d^	1490 ± 210 ^a^	≥90 ^a^
15% Whey + 5% SM	82.03 ± 4.86 ^c^	1038 ± 83 ^b^	≥90 ^a^
10% Whey + 10% OM	22.61 ± 4.25 ^d^	222 ± 39 ^cd^	≥90 ^a^
10% Whey + 10% SM	9.75 ± 1.26 ^d^	172 ± 25 ^cd^	≥90 ^a^

Different parameter superscripts (a–d) in the same column indicate significant differences (*p* < 0.05).

**Table 2:** Texture profile analysis (TPA) parameters of whey protein and whey protein-mushroom samples with different compositions were determined using a double compression-decompression test. All samples contained 100 mM NaCl. Samples with different letters are significantly different (*p* < 0.05) in the same column (same TPA parameter).

Group	Hardness (g)	Adhesiveness	Resilience (%)	Cohesion	Springiness (%)	Gumminess	Chewiness
20% Whey	14,600 ± 1300 ^a^	−0.03 ± 0.05 ^a^	15.17 ± 0.81 ^b^	0.35 ± 0.04 ^c^	94.5 ± 6.5 ^a^	49.7 ± 4.2 ^b^	47.1 ± 6.5 ^b^
15% Whey	5400 ± 970 ^c^	−0.19 ± 0.12 ^a^	14.1 ± 1.3 ^b^	0.35 ± 0.04 ^c^	81.2 ± 7.3 ^ab^	18.7 ± 4.1 ^cd^	15.3 ± 4.3 ^cd^
10% Whey	1330 ± 340 ^d^	−0.24 ± 0.13 ^a^	13.6 ± 1.8 ^b^	0.35 ± 0.03 ^c^	64.7 ± 24.5 ^abc^	4.7 ± 1.6 ^d^	3.0 ± 1.2 ^d^
15% Whey + 5% OM	14,200 ± 1700 ^a^	−6.09 ± 2.4 ^b^	22.84 ± 0.48 ^a^	0.58 ± 0.03 ^a^	81.2 ± 1.3 ^ab^	81 ± 11 ^a^	65.8 ± 9.0 ^a^
15% Whey + 5% SM	10,300 ± 1400 ^b^	−5.93 ± 0.91 ^b^	14.2 ± 1.3 ^b^	0.47 ± 0.01 ^b^	58.9 ± 7.3 ^bc^	47.6 ± 7.4 ^b^	28.4 ± 7.7 ^c^
10% Whey + 10% OM	7840 ± 910 ^bc^	−2.65 ± 0.94 ^a^	12.4 ± 1.4 ^b^	0.38 ± 0.01 ^c^	47.6 ± 3.9 ^cd^	28.9 ± 3.0 ^c^	13.8 ± 2.0 ^cd^
10% Whey + 10% SM	1850 ± 240 ^d^	−2.16 ±0.43 ^a^	6.29 ± 0.47 ^c^	0.33 ± 0.04 ^c^	18.75 ± 3.63 ^d^	5.85 ± 0.54 ^d^	1.08 ± 0.12 ^d^

## Data Availability

The raw data supporting the conclusions of this article will be made available by the authors on request. Additional inquiries should be addressed to the corresponding author.
